# Human Umbilical Cord Mesenchymal Stem Cell Derived Exosomes Delivered Using Silk Fibroin and Sericin Composite Hydrogel Promote Wound Healing

**DOI:** 10.3389/fcvm.2021.713021

**Published:** 2021-08-19

**Authors:** Chaoshan Han, Feng Liu, Yu Zhang, Wenjie Chen, Wei Luo, Fengzhi Ding, Lin Lu, Chengjie Wu, Yangxin Li

**Affiliations:** Department of Cardiovascular Surgery, Institute for Cardiovascular Science, Collaborative Innovation Center of Hematology, First Affiliated Hospital and Medical College of Soochow University, Suzhou, China

**Keywords:** silk fibroin and sericin composite hydrogel, exosome, wound repair, angiogenesis, inflammation

## Abstract

Recent studies have shown that the hydrogels formed by composite biomaterials are better choice than hydrogels formed by single biomaterial for tissue repair. We explored the feasibility of the composite hydrogel formed by silk fibroin (SF) and silk sericin (SS) in tissue repair for the excellent mechanical properties of SF, and cell adhesion and biocompatible properties of SS. In our study, the SF SS hydrogel was formed by SF and SS protein with separate extraction method (LiBr dissolution for SF and hot alkaline water dissolution for SS), while SF-SS hydrogel was formed by SF and SS protein using simultaneous extraction method (LiBr dissolution for SF and SS protein). The effects of the two composite hydrogels on the release of inflammatory cytokines from macrophages and the wound were analyzed. Moreover, two hydrogels were used to encapsulate and deliver human umbilical cord mesenchymal stem cell derived exosomes (UMSC-Exo). Both SF SS and SF-SS hydrogels promoted wound healing, angiogenesis, and reduced inflammation and TNF-α secretion by macrophages. These beneficial effects were more significant in the experimental group treated by UMSC-Exo encapsulated in SF-SS hydrogel. Our study found that SF-SS hydrogel could be used as an excellent alternative to deliver exosomes for tissue repair.

**Graphical Abstract:**
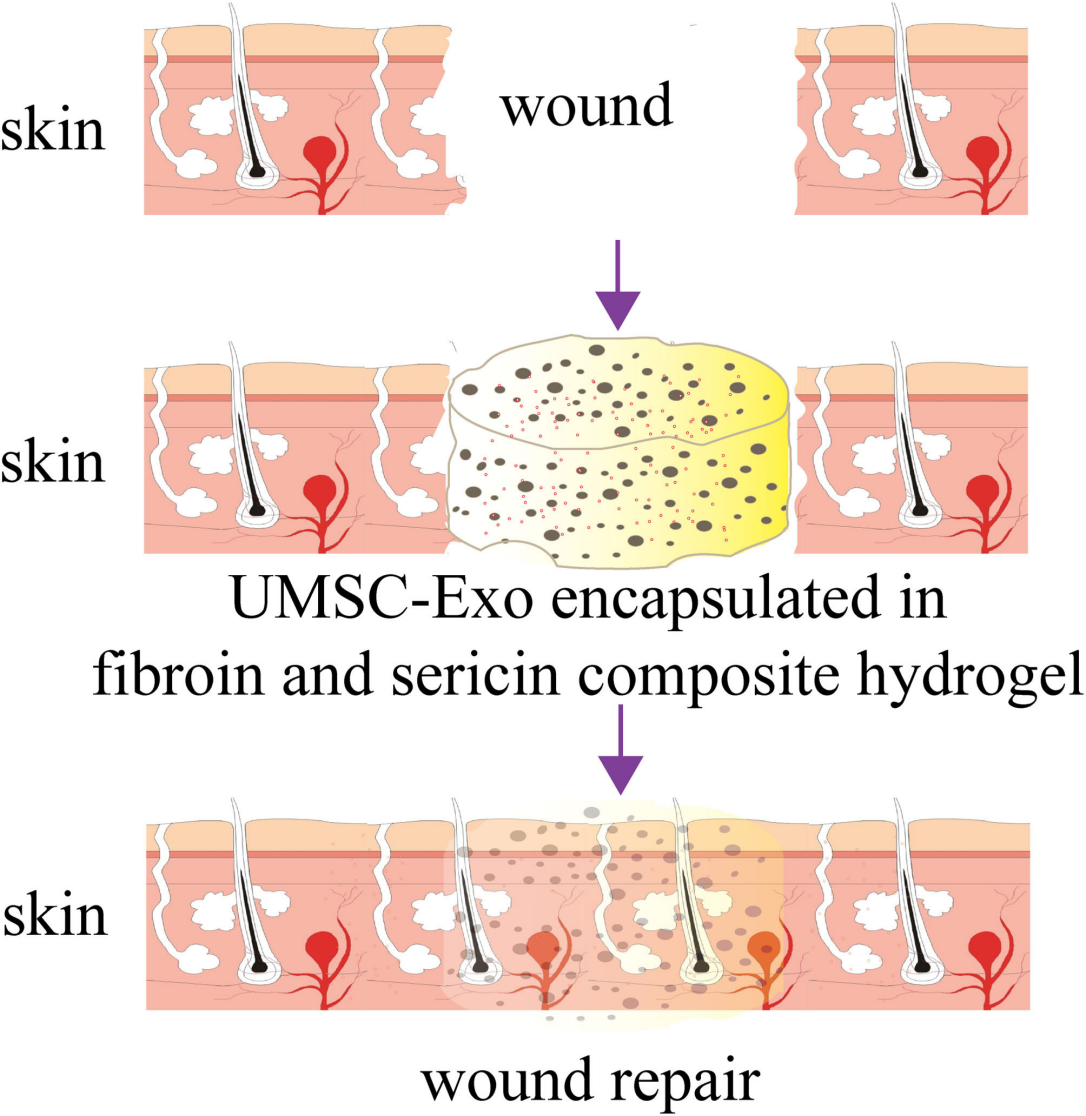
The composite hydrogel formed by SF and SS did not induces strong immune responses and improved the beneficial effect of the UMSC-Exo by promoting angiogenesis and the wound repair. The composite hydrogel can serve as an excellent media for exosomes delivery and tissue repair.

## Introduction

As the biggest organ, skin functions as a barrier to protect the body from pathogen invasion and to prevent fluid loss. However, skin often suffers from injuries caused by burns, diabetes, and ulceration, which cause infections, as well as physical and mental pain of the individual (1). Many ongoing research efforts have been focused on developing methods to promote wound healing. Those method should keep the wound from being infected and allow gas exchange, adsorb excrescent wound exudates and maintain the local moisture of the wound to accelerate the healing (2). Recently, stem cell and exosomes have emerged as promising strategies for skin repair. Exosomes are 30–100 nm extracellular vesicles that can be secreted by almost all cells, and stem cell-derived exosomes have been shown to accelerate the healing of skin wound (3). Exosomes derived from the human umbilical cord mesenchymal stem cells (UMSC-Exo) have been shown to promote re-epithelialization, enhance collagen maturation and reduce the scar size of skin wound. In addition to UMSC-Exo, exosomes secreted by pluripotent stem cells-derived MSCs, intestinal epithelial cells, oral mucosal epithelial cell, adipose stem cells, induced pluripotent stem cells and platelet (3–8) could also promote skin wound healing. Although these previous studies proved the concept of principal, there are still hurdles that need to be overcome before exosome-based therapy can be used in the clinic. Exosomes have short retention time *in vivo* due to the lack of matrix support. In addition, exosomes often present in solution, which is not able to protect the wound from the pathogenic bacteria, and not absorb excrescent exudates, so exosomes must be delivered together with wound dressing materials to achieve the desired beneficial effects.

Hydrogels have been used as excellent wound dressing (9, 10). Hydrogels can be formed by natural, synthetic or composite materials. Single material-based hydrogel has some drawbacks for the lack of either biological function or good mechanical properties, whereas the composite hydrogel formed by two or more materials can avoid these defects. Silk, an appealing biomaterial that was widely used in medical field, is consisted of 70% silk fibroin (SF), 25% Silk sericin (SS). SF has been used as suture thread and wound dressing for its good biocompatibility and suitable mechanical properties (11–15). The biological properties of SF can be improved when introduce other materials such as chitosan, polyurethane, hyaluronic acid (15–18). Even though SS was originally thought to trigger immune response and was treated as the by-product in traditional silk biomaterial processing (19). Recent studies have shown that SS is biocompatible and has good biological performance. SS shows great hydrophilic and has multiple biological properties, including anti-oxidation, anti-bacterial, and promoting cell adhesion and proliferation (20, 21). However, SS shows poor mechanical properties. It was shown that the Young's moduli of SS hydrogel are much lower than that of most tissues (22). Thus, SS has been mostly used in tissue regeneration and wound repair in combination with other materials (10, 23, 24). It's an interesting find that SF has an opposite characteristic that Young's moduli of SF hydrogel have a higher stiffness than native tissue (25).

Thus, introducing SS into the fibroin hydrogel can adjust the compressive moduli to a more suitable condition for tissue engineering. More importantly, sericin has the beneficial characteristics to promote cell adhesion and growth (26). In addition, the different hydrophilic and hydrophobic nature of sericin and fibroin of the complex hydrogel enables sericin to be exposed to the water, which makes it easier for sericin to interact with surrounding tissue and to exert its beneficial effect.

Thus, in our study, we explored the immunogenicity of the composite hydrogel formed by SF and SS and determined whether the composite hydrogel can deliver UMSC-Exo to repair skin wound.

## Materials and Methods

### Isolation and Identification of Exosomes

The exosomes derived from the human umbilical cord mesenchymal stem cells (UMSCs) were isolated and collected as described previously (27, 28). Briefly, UMSCs (Jiangsu Heze Biotechnology, Heze, Jiangsu, China) were cultured in MEM medium containing 15% FBS and cells of passage 4–6 were digested by 0.125% (w/v) trypsin (Thermo fisher, Waltham, MA, USA) and the cell suspension was transferred to a new 10 cm cell culture dish, until reaching to 70% confluency and was then replaced by MEM medium containing 10% FBS which had been centrifuged at 100,000 g to eliminate bovine-derived exosomes. After 48 h of culture, exosomes were isolated from UMSCs culture supernatants which was centrifuged at 2,000 g for 30 min to remove dead cells and debris, and then centrifuged at 10,000 g for 40 min to remove large extracellular vesicles, then the medium was transferred to a new tube containing 0.5 volumes of Total Exosome Isolation reagent (Life Technology, Grand Island, NY, USA). The mixture was incubated at 4°C overnight and centrifuged at 10,000 g for 1 h at 4°C. The pellets were re-suspended in PBS and stored at −80°C. Protein concentrations of UMSC-Exo were determined using a BCA protein assay kit (Takara, Japan).

The diameter of UMSC-Exo was analyzed by Zetasizer Nano (Malvern Panalytical, Malvern, UK). Exosomes (1 mL) with a concentration of 1 mg/mL were placed in a quartz colorimetric dish for subsequent measurement. Specific protein markers of exosomes were identified by Western blot and flow cytometry as we reported previously (27, 28). Briefly, 40 μg protein extracted from UMSC-Exo or UMSCs protein was separated by SDS-PAGE, transferred to PVDF membranes which were incubated with CD63 (Santa cruz biotechnology, Texas, USA), CD9 (Abcam, Cambridge, MA, USA), Glyceraldehyde 3-phosphate dehydrogenase (GAPDH) (MultiSciences, Hangzhou, China) antibodies, followed by washing and incubation with HRP-linked goat anti-rabbit IgG (MultiSciences, Hangzhou, China) and HRP-linked goat anti-mouse IgG secondary antibodies (MultiSciences, Hangzhou, China). Exosomes were attached to aldehyde/sulfate latex beads (4 μm; Invitrogen, Grand Island, NY, USA) for analysis. The pre-bound exosomes were analyzed by flow cytometry using a fluorescence isothiocyanate-conjugated antibody against CD63 (Abcam, Cambridge, MA, USA), which is a specific marker for exosomes.

### Preparation of SF SS Hydrogel

The separate extraction of SF and SS (SF SS) from silkworm (*Dazao* strain) cocoons were performed as described previously. Briefly, 5 g silkworm cocoons were cut into 1 cm^2^ pieces and boiled in 1 L 0.02 M Na_2_CO_3_ solution (Macklin, Shanghai, China) for 30 min with continuous stirring in order to disperse SF and allow SS to be dissolved. SS was obtained by concentration and dialysis of the harvested solution. The SS solution was finally adjusted to 5% w/v with ultrapure water at room temperature. After washing in ultra-pure water to remove the remaining SS protein, the drying SF was extracted as described before (29). Briefly, the SF was dissolved in 9.3 M LiBr (Thermo Scientific, Waltham, MA, USA) and SF solution was load in ordinary dialysis bag (YuanyeBio-Technology, Shanghai, China) with cut-off molecular weight of 3,500 and dialyzed in ultrapure water for 48 h, then centrifuged to remove precipitates. The SF solution was finally adjusted to 5% (w/v) with ultrapure water and stored at 4°C for up to one week. The concentration of SF or SS solution was determined by the measuring dry weight of SF or SS after drying the entire content of the solution. In order to ensure the similar proportion of SF and SS in SF SS and SF-SS hydrogel, we measured the SF and SS in natural silk, the dried weight of SF was 3.71g after removing the SS from 5 g silk, so the content of fibroin was 74.2% (3.71 g ÷ 5 g × 100% = 74.2%). Considering the fact that there is about 3.2% carbohydrates, inorganic matter and pigment dissolved in the SS solution, the content of sericin in silk is 22.6% (100%−74.2%−3.2% = 22.6%). The proportion of SF to SS is about 3:1 in natural silk, which is similar to that of SF-SS hydrogel. The volume ratio of SF solution to SS solution was 3:1, which is consistent with that of the natural silk. The mixed solution (0.5 ml) was sonicated 3 times for 30 s at 30% ultrasound intensity (S150D, Branson, St. Louis MO, USA), and then the pre-gelling SF SS mixed solution was transferred to the molds with a diameter of 1.2 cm to ensure the hydrogel has a height of 3 mm, and then kept at 37°C for 24 h to form SF SS hydrogel.

### Preparation of SF-SS Hydrogel

The simultaneous extraction of SF and SS (SF-SS) from silkworm cocoons were performed using a LiBr dissolution as previously described (30). Briefly, 5 g silkworm cocoons were cut into 1 cm^2^ pieces and was dissolved in 9.3 M LiBr to make a 20% (w/v) protein solution and then incubated at 60°C to completely dissolve the SF and SS. After dialysis and concentration, the SF-SS solution was finally adjusted to 5% (w/v) with ultrapure water at room temperature and stored at 4°C for no more than one week. The mixed SF-SS protein solution was sonicated 1 times for 30 s at 30% ultrasound intensity, and then the pre-gelling SF-SS solution was transferred to the suitable molds, and kept at 37°C for 1 h to form the SF-SS hydrogel.

### Exosomes Encapsulated Into the SF SS or SF-SS Hydrogel

SF SS hydrogel or SF-SS hydrogel were frozen in −20°C freezer, and then freeze-dried for 48 h in freeze dryer to generate hydrogel sponge. Exosomes derived from UMSCs were isolated and the concentration was measured as described in 2.1. Exosomes concentration would be adjusted to 2 mg/mL and 50 μL exosomes will be dropped into the hydrogel sponge and it will be used for wound repair immediately. The hydrogel sponge lost most of water and it will hold the exosome solution.

### Cell Proliferation Assay

The human skin fibroblast cells (BJ cells) were purchased from ATCC (Maryland, USA). The BJ cells were cultured in DMEM to reach 70–80% confluency in 96-well plates and then switched to fresh DMEM. UMSC-Exo (100 or 200 μg), 5 μg freeze-dried SF SS hydrogel, 5 μg SF-SS hydrogel or sterile cotton gauze were added into different wells and then incubated with BJ cells for another 24 h. After removing the residual cotton gauze or the freeze-dried hydrogel, cell viability was determined using the CCK-8 reagent (MesGen Biotech, Shanghai, China) per manufacturer's instruction. In order to observe the cells proliferation directly, a white pipette tip was used to draw a straight line on the monolayer cells to create an open space when the BJ cells were cultured to reach 85–90% confluency in 96-well plates. The cells were washed 3 times with PBS to remove free floating cells formed by scratches. The sterile gauze or the freeze-dried SF SS and SF-SS hydrogel were added into the DMEM medium with 10% FBS and incubated with BJ cells. After 24 h co-culture, the cells were fixed and photographed under a microscope.

### Fluorescence and Microstructure of Silk Fibroin and Sericin Mixed Hydrogel

In order to clearly observe the fluorescence characteristics of different hydrogel under a microscope, the thickness of the hydrogel was controlled to 0.5–1 mm. After freeze-drying, the spontaneous fluorescence under different fluorescence channels of an inverted fluorescence microscope is observed directly and recorded.

To reveal the morphology of the SF SS and SF-SS hydrogel, the samples were loaded on top of conductive tapes mounted on SEM sample stubs and sputter-coated with gold for 60 s using gold sputter-coating equipment (Cressington Scientific Instruments, Watford, UK). The samples were examined using a scanning electron microscope (S-4800; Hitachi, Japan) at an accelerating voltage of 3 kV.

### Attenuated Total Reflection-Fourier Transform Infrared Spectroscopy (ATR-FTIR) Measurements

A VERTEX 70 FTIR spectrometer (Bruker, Hongkong, China) equipped with a diamond ATR accessory, a deuterated triglycine sulfate detector, and a KBr beam splitter was used for spectral acquisition. The freeze-dried samples were then placed on the diamond ATR crystal. For each sample, three replicate spectra were recorded to ensure the spectral reproducibility and analytical precision. All spectra were recorded in the range of 4,000–600 cm^−1^ using the ATR method with a resolution of 4 cm^−1^ and 32 scans.

### Compressive Mechanical Property Testing

The SS and SF mixed solution was sonicated and loaded into the suitable mold to form a cylinder hydrogel with the diameter of 120 mm and the height of 150 mm. After freeze drying, hydrogel were tested by a universal material testing machine at room temperature. In compression test, the loading speed was 5 mm/min. When the sample was compressed to 60% of its original height, the loading was stopped. Each sample was tested three times.

### Wound Closure Assay

Before the surgery, the C57BL/6J mice were anesthetized by intraperitoneal injection of sodium pentobarbital (45 mg/kg). After shaving the dorsal hairs and sterilizing the skin, a 10 mm diameter full-thickness wound was created on the upper back. The mice were randomly divided into five groups (*n* = 6/group). Control group: four layers cotton gauze (13 × 13 mm) containing 50 μL PBS were inserted into the skin layer near the skin defect to cover the wound. SF SS or SF-SS Hydrogel group: a freeze-dried hydrogel (13 × 13 mm) containing 50 μL PBS was used to cover the wound as the described in the control group. SF SS Hydrogel-Exosome or SF-SS Hydrogel-Exosome group: a freeze-dried hydrogel containing 100 μg exosomes in 50 μL PBS was used to cover the wound. The restraining bandage (Urgostrapping, URGO) was used to fix the wound and dressings. At 3 and 21 days post-surgery, 3 mice in each group were sacrificed for further analysis. The mice were maintained in a specific pathogen-free animal facility at Soochow University, and fed with sterile food and water. All animal experiments were carried out in accordance with the National Institutes of Health Guide for the Care and Use of Laboratory Animals (NIH Publications No. 8023, revised 1978). The animal protocols were approved by the Laboratory Animal Care and Use Committee of Soochow University.

### Immunostaining for CD31 and CD68 Expression

To avoid skin contraction, complete wound specimens and the normal skin tissue within 2–3 mm around the wounds were collected on days 3 and 21 post-operation. The skin tissues were fixed overnight by 4% polyoxymethylene and then dehydrated by 20% sucrose. After embedding in optimal cutting temperature compound and freezing in liquid nitrogen, the specimens were cut into 5-μm sections for immunostaining. After blocking with 5% BSA, skin sections were incubated with a mouse anti-CD31 antibody (Abcam, Cambridge, MA, USA) or anti-CD68 antibody (Abcam), followed by a goat anti-mouse IgG-FITC secondary antibody (Santa Cruz Biotechnology, Texas, USA). The nuclei were stained with DAPI (Sigma-Aldrich, St. Louis, MO USA). Images were analyzed using fluorescent microscopy.

### TNF-α Detection by ELISA

The macrophage NR8383 cells (Kang Lang Biological Technology, Shanghai, China) were used to assess the immune response induced by the SF SS or SF-SS hydrogel sponges. 2 × 10^5^ NR8383 cells were seeded into each well of the plate 24-well plate. When the cells reached 75–85% confluency, freeze-dried SF SS, SF-SS hydrogel or cotton gauze were added into culture media and co-culture for 24 h, and then the culture media was harvested and centrifuged at 1,000 rpm for 5 min. TNF-α levels were detected by TNF-α ELISA kit (Abcam).

Blood samples were collected from the animals and the blood samples were placed at 37°C for 1 h and sera were obtained by centrifugation at 5,000 rpm for 5 min. The levels of TNF-α were determined by ELISA kit.

### Statistical Analysis

All data with significance of differences were presented as mean ± standard deviation. Statistical analyses and calculation of sample size were performed using Prism 5 software (GraphPad Software, La Jolla, CA, USA). Two-tailed *t*-test was used to determine the significance of differences between two groups. Multiple comparisons were analyzed using ANOVA with post-*hoc* analysis by the Newman-Keuls test.

## Results

### Isolation, Identification and Function of Exosomes Derived From Human Umbilical Cord Mesenchymal Stem Cells (UMSC-Exo)

**Figure 1:**
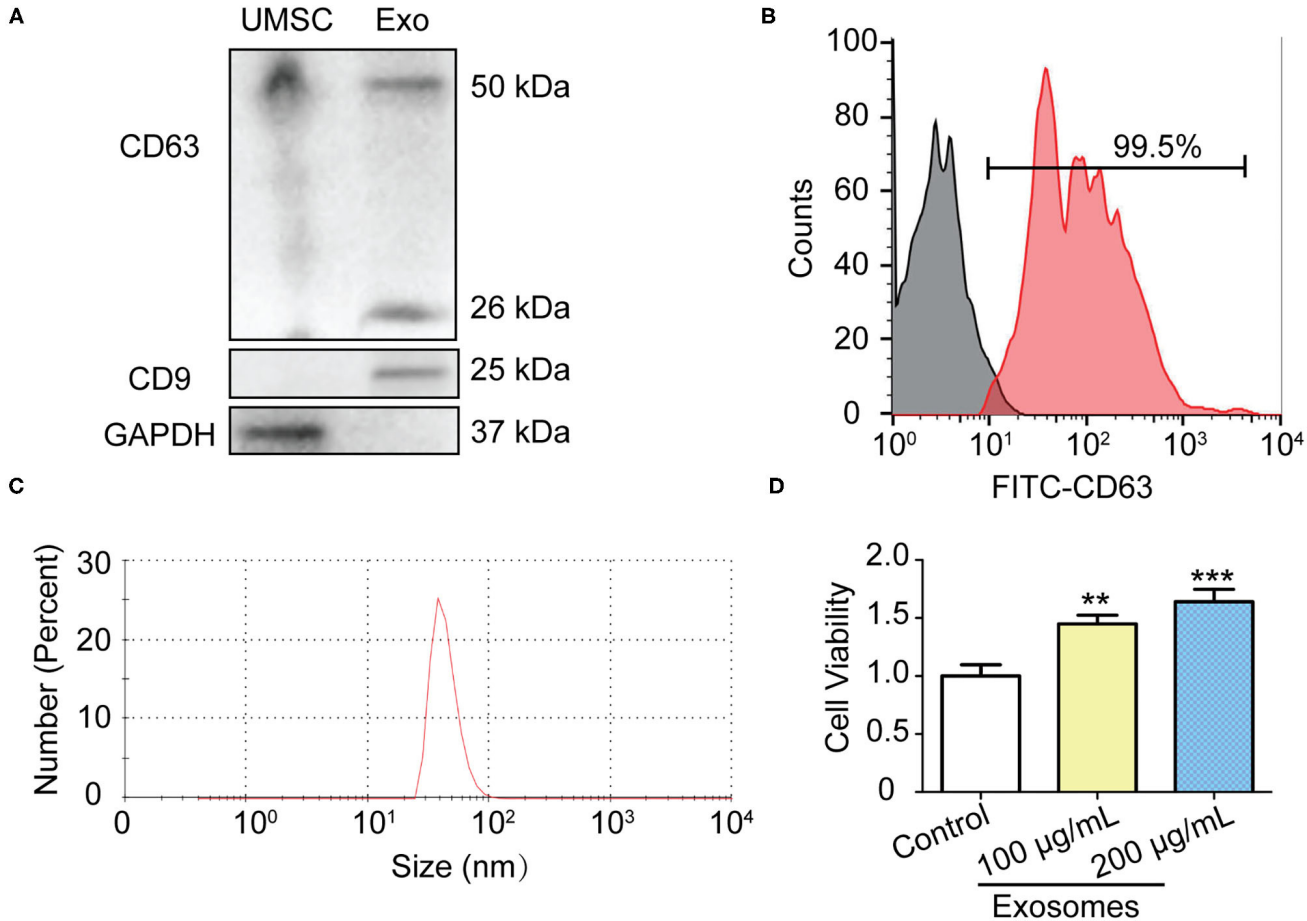
Isolation and identification of UMSC-Exo. **(A)** Western blot analysis of CD9 and CD63 and GAPDH protein expression in UMSC and UMSC-Exo. **(B)** The expression of CD63 in UMSC-Exo was analyzed by flow cytometry. **(C)** Nanoparticle size distribution of UMSC-Exo. **(D)** The BJ cell viability was detected by CCK-8 after treatment with UMSC-Exo. Data is shown as mean ± standard deviation (*n* = 3). ^**^p < 0.01, ^***^p < 0.001.

In order to obtain pure and well-characterized exosome preparations, we used the commercial ready-to-use precipitation solutions and total exosome isolation reagent to separate exosomes from supernatants of cell culture. Cell debris and large vesicles were removed by centrifugation. The exosomes were characterized by Western blot, and Zetasizer Nano analysis. Western blot showed that GAPDH was highly expressed in the UMSC, whereas exosomes-specific markers CD9 and CD63 were only detected in UMSC-Exo (Figure 1A). Flow cytometry results further proved that more than 99% of the exosome-coated beads are CD63 positive (Figure 1B). We also found that CD63, which is a universal biomarker of exosomes, has a higher expression in UMSC-Exo. The diameter of exosomes was approximately 47.3 nm determined by Zetasizer Nano (Figure 1C), which is consisted with the previous reported value of 30–100nm. In addition, the cell viability of skin fibroblast was significantly enhanced in a dose dependent way when incubated with the exosomes with the concentration of 100 and 200 μg/ml (Figure 1D), which is consistent with previous study (31).

### Silk Fibroin and Sericin Mixed Hydrogel Increase Skin Fibroblast Cell Viability and Proliferation

**Figure 2:**
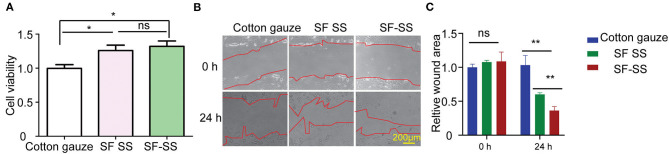
SF SS and SF-SS hydrogel promote BJ cell growth. **(A)** BJ cell viability was detected by CCK-8 after treatment with SF SS and SF-SS hydrogel. **(B)** BJ cell migration after scratch and treatment with SF SS and SF-SS hydrogel was recorded by microscope. **(C)** The quantity of relative wound area in **Figure B** of cell migration by Image J. Data is shown as mean ± standard deviation (*n* = 3). ns, no significant difference, ^*^p < 0.05, ^**^p < 0.01.

SF SS hydrogel was formed by SF and SS protein with separate extraction method (mild LiBr dissolution for SF and high temperature alkaline water method for SS), while SF-SS hydrogel was formed by SF and SS protein using simultaneous extraction method (mild LiBr dissolution for SF and SS protein). When SF SS and SF-SS hydrogel was incubated with the BJ cells, the cell viability was increased significantly (Figure 2A). Moreover, the cell doubling time is fast in SF-SS hydrogel with mild extraction method (Figures 2B,C), which may be due to the relative mild extraction process for protein in SF-SS hydrogel and more intact bioactive SS can be preserved during extraction process of mild LiBr dissolution method.

### Characterization of Silk Fibroin and Sericin Mixed Hydrogel

**Figure 3:**
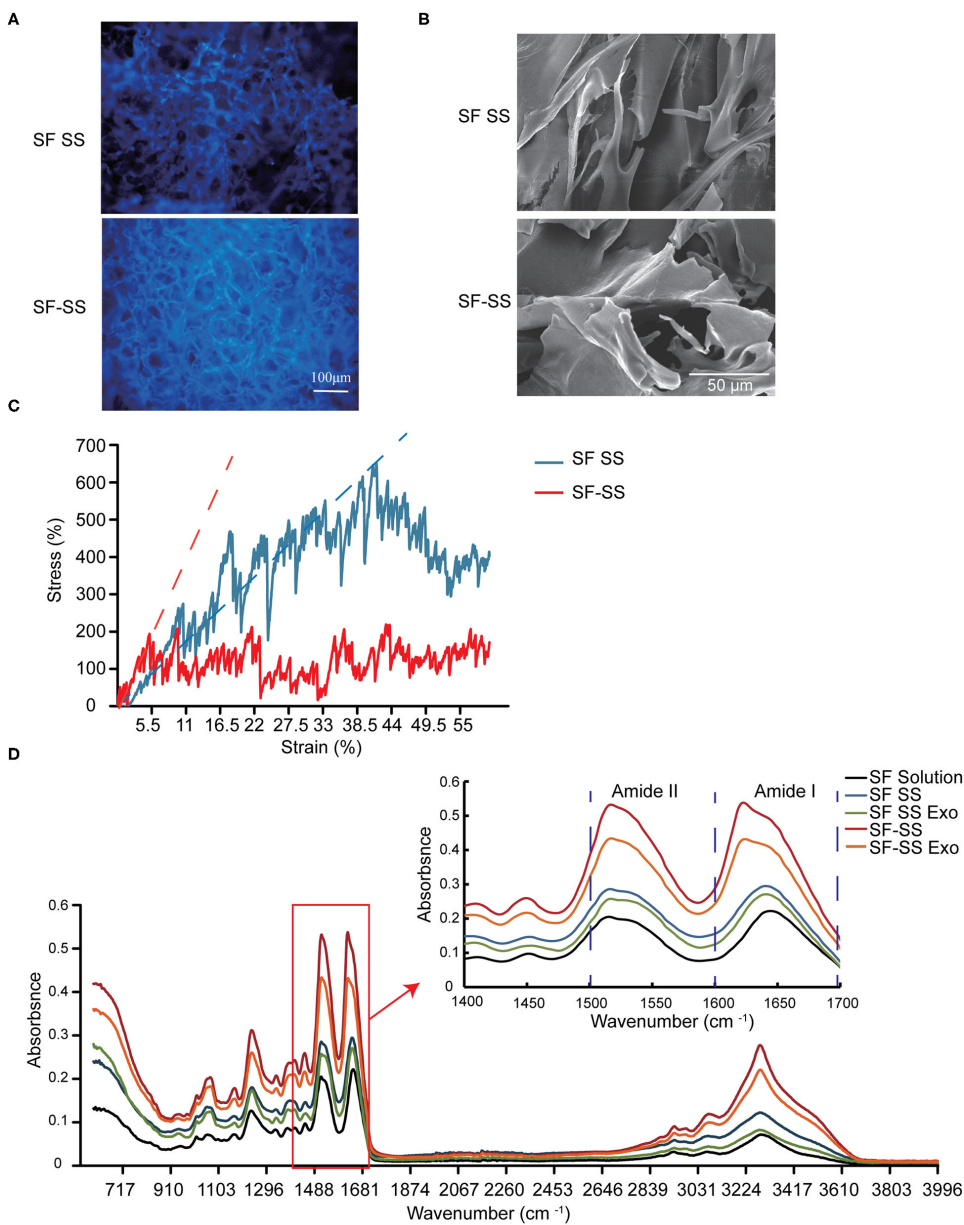
Characterization of SF SS and SF-SS hydrogel. **(A)** Spontaneous fluorescence and microstructure of SF SS and SF-SS hydrogel. **(B)** Microstructure of SF SS and SF-SS hydrogel. **(C)** The stress-strain curve of SF SS and SF-SS hydrogel. **(D)** Fourier transform infrared spectroscopy of SF SS and SF-SS hydrogel.

Hydrogel formed by fibroin and sericin can emit intrinsic fluorescence (Figure 3A), which is partly due to 5% tyrosine and 0.25% tryptophan in fibroin, 2.1% tyrosine and 0.9% phenylalanine in sericin (32). SF-SS hydrogel shows a stronger spontaneous fluorescence and a smaller pore diameter compared to SF SS hydrogel (Figures 3A,B). The pore size of hydrogel is about 20–100 μm as shown in Figures 3A,B, which is much larger than exosomes with a diameter of 30–100 nm. This result suggests that most of exosomes can be diffused into the surrounding environment. The Young's moduli were 1.56 kPa (SF SS hydrogel) and 3.77 kPa (SF-SS hydrogel), which was reflected by straight line slope as shown in Figure 3C. Thus, compressive mechanical property revealed that the SS SF hydrogel had a higher stress than the SS-SF hydrogel (Figure 3C). The Young's moduli of SF SS hydrogel and SF-SS hydrogel indicated that the complex hydrogels are suitable for the usage on the soft tissue.

FTIR analysis revealed a shift to lower wavelength at the peak of Amide I and Amide II in SF SS hydrogel and SF SS Exo hydrogel compared with the SF solution, and the wavelength became even lower in SF-SS hydrogel and SF-SS Exo hydrogel, suggesting the formation of a more stable β-folding structure (Figure 3D). Adding exosome caused only a small shift to lower wavelength in both SF SS hydrogel and SF-SS hydrogel. The significant difference between SF SS hydrogel and SF-SS hydrogel may be attributed to the mild simultaneous protein extraction conditions of SF-SS hydrogel to allow easier formation of hydrogel.

### The Effect of Silk Fibroin and Sericin Mixed Hydrogel on Wound Closure

**Figure 4:**
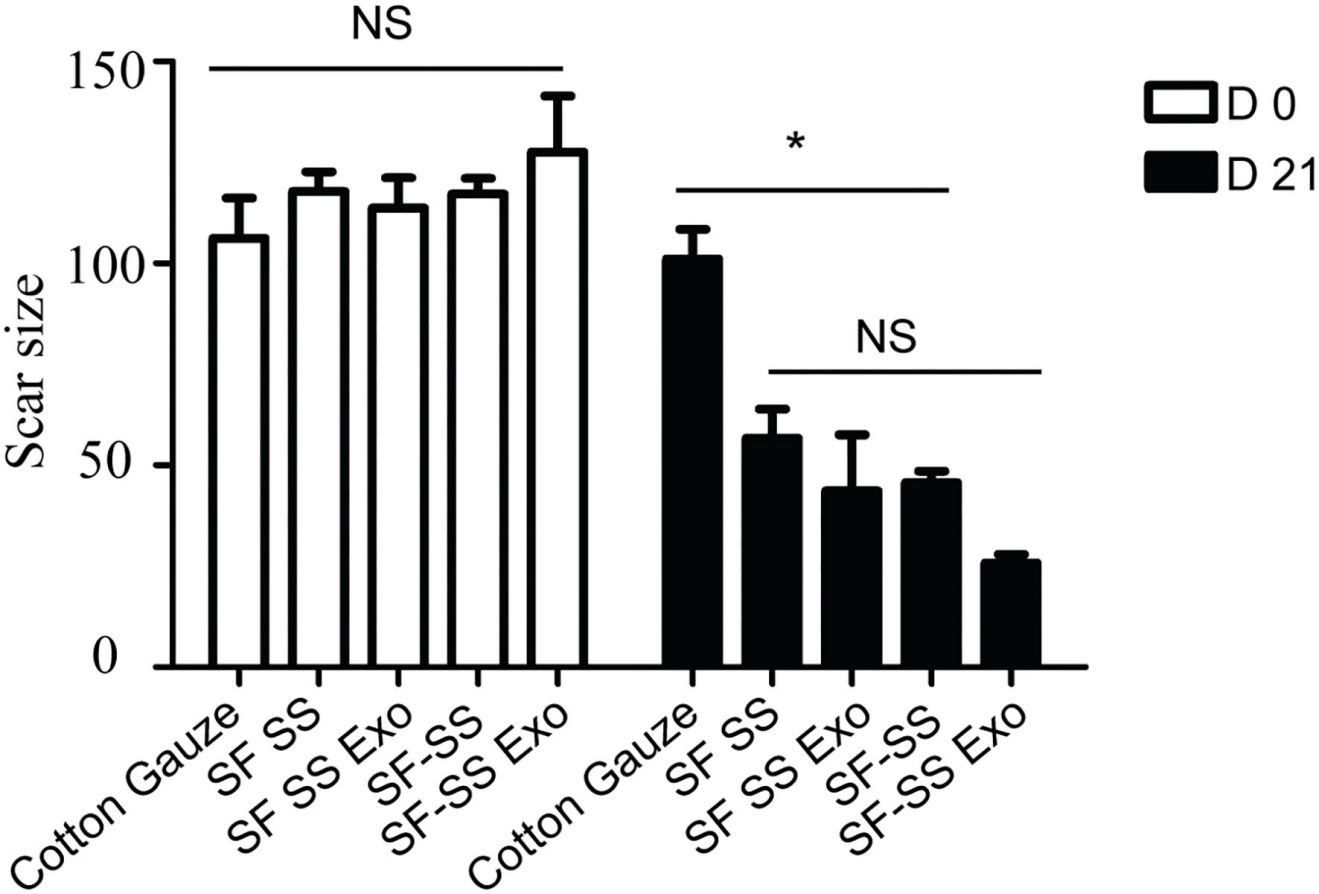
The wound closure ability of SF SS and SF-SS hydrogel. The scar size analysis by Image J. Data is shown as mean ± standard deviation (*n* = 3). ns, no significant difference, ^*^p < 0.05.

**Figure 5:**
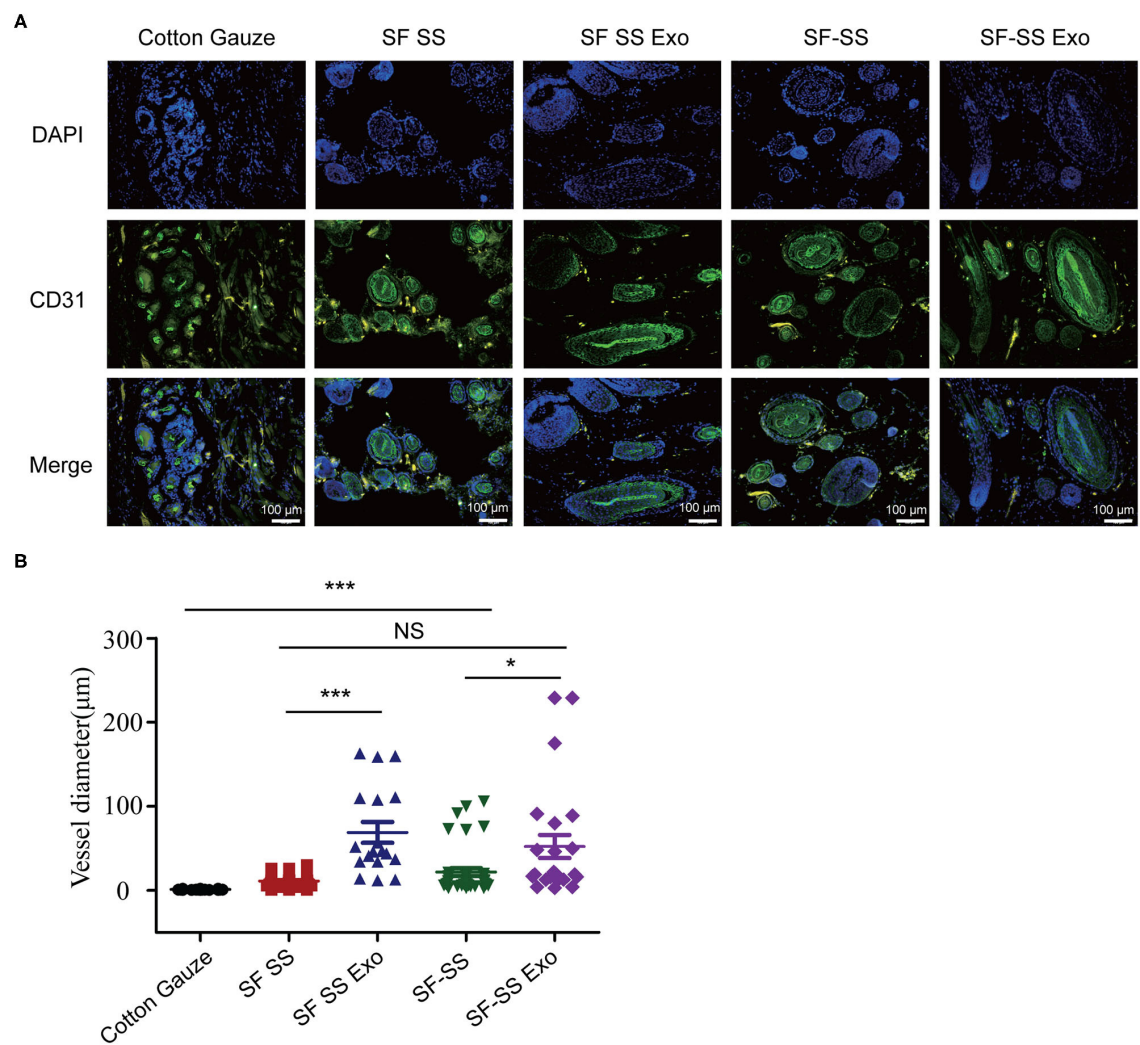
The immunogenicity of SF SS and SF-SS hydrogel sponges. **(A)** Immunofluorescence staining of CD31 in skin wound treated by SF SS and SF-SS hydrogel and UMSC-Exo encapsulated in SF SS and SF-SS hydrogel. SF SS Exo, the Exo was delivered by SF SS composite hydrogel; SF-SS Exo, the Exo was delivered by SF-SS composite hydrogel. **(B)** Quantification of vessel diameter by Image J. Data is shown as mean ± standard deviation (*n* = 3). ns, no significant difference, ^*^p < 0.01, ^***^p < 0.001.

Mice were created a 10 mm diameter full-thickness wound on the upper. After inserting the cotton gauze or hydrogel sponge into the wound, the wound was fixed with restraining bandage and the wound repairment would be observed. Three days after treatment, the two composite hydrogels absorbed most of the wound exudates and maintain the moisture environment, which was favorable to wound healing. However, the gauze induced significant exudates that caused the gauze to stick to the tissue and slowed down the healing process. After 21 days treatment, the skin cells can grow on the surface of the composite SF SS and SF-SS hydrogel and the wound was completely healed, whereas the size of the gauze inserted skin wound remains unhealed. In the presence of exosomes, SF SS and SF-SS not only healed the wounds completely but also recovered the hair growth to normal level (Figure 4). CD31 staining on skin wound sections was used to assess angiogenesis. Even though density of total vessels in gauze cotton was more than that of other treatment, most of vessels showed a non-mature state in gauze cotton. Thus, we mostly analyzed the vessels diameter in different groups. The blood vessels were longer in the SF SS Exo and SF-SS Exo groups compared with the control, SF SS and SF-SS groups (Figures 5A,B). In addition, the scar size in SF-SS group is smaller that of SF SS group (Figure 4), and the average diameter of blood vessels is larger than that of SF SS group (Figure 5B).

### The Inflammatory Response to Silk Fibroin and Sericin Mixed Hydrogel *in vitro* and *in vivo*

**Figure 6:**
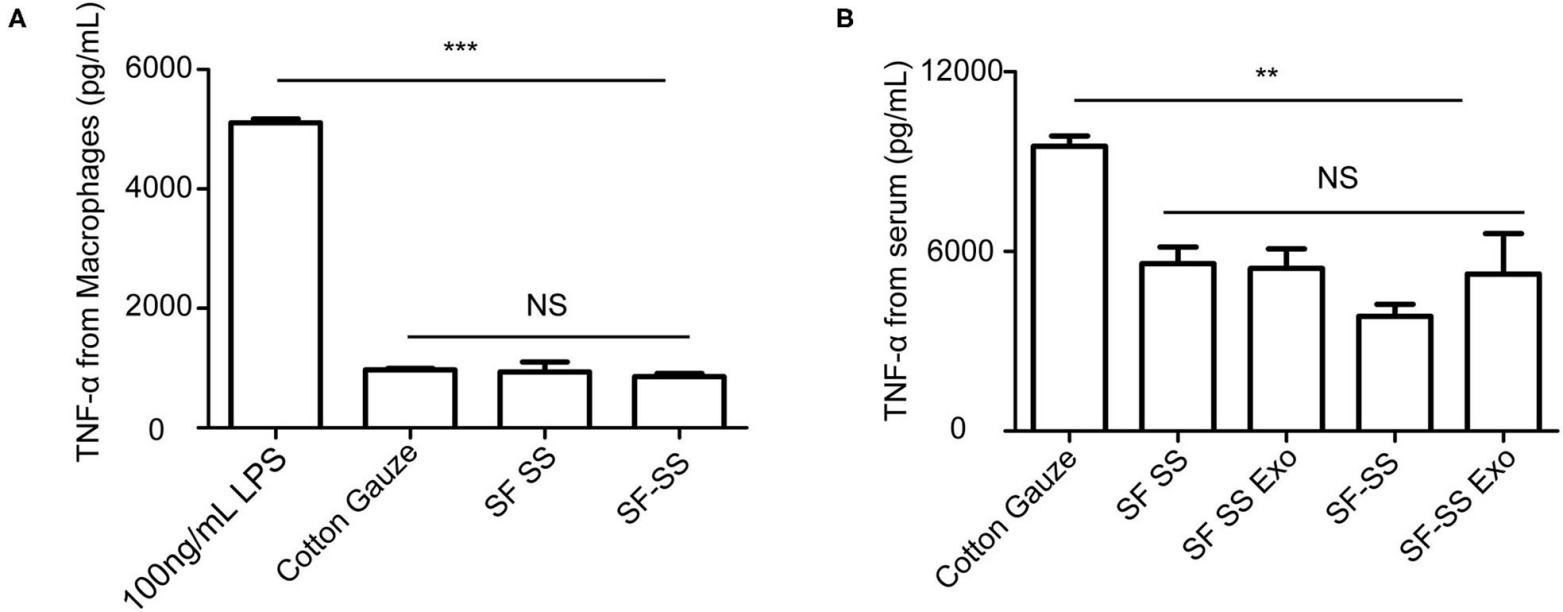
TNF-α released by macrophage or in serum after incubating with SF SS and SF-SS hydrogel sponges. **(A)** TNF-α release from macrophages after incubating with cotton gauze, SF SS and SF-SS hydrogel. **(B)** TNF-α in the serum from mice with different treatments. Data is shown as mean ± standard deviation (*n* = 3). ns, no significant difference, ^*^p < 0.05, ^**^p < 0.01, ^***^p < 0.001.

**Figure 7:**
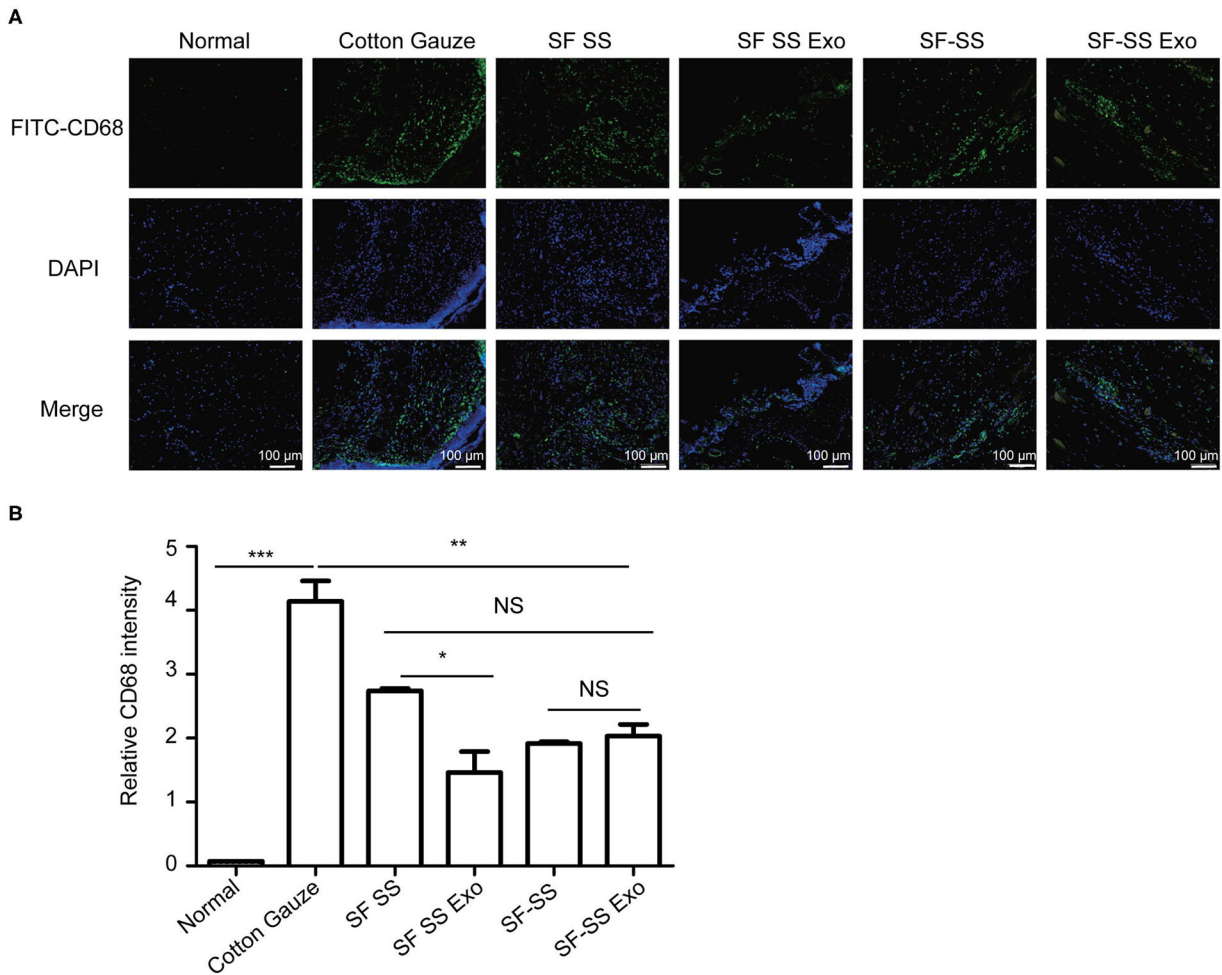
CD68^+^ cells infiltration in wound treated with SF SS and SF-SS hydrogel sponges. **(A)** CD68^+^ inflammatory cell infiltration in skin wound from different treatment groups. **(B)** Quantification of CD68^+^ cells with the FITC fluorescence signal by Image J. Data is shown as mean ± standard deviation (*n* = 3). ns, no significant difference, ^*^p < 0.05, ^**^p < 0.01, ^***^p < 0.001.

To determine whether the hydrogel induces inflammation, the SF SS and SS-SF hydrogel sponges were incubated with macrophages in cell culture for 24 hours or applied to the skin wound of a mice model for 3 days. The cell culture supernatant or serum from the mice was collected to measure the level of TNF-α. Previous studies have suggested that SF and SS can induce inflammation; however, there is no significant difference in the amount of TNF-α released from either the macrophages or mice with injured skin (Figures 6A,B). *in vitro*, the amount of TNF-α derived from macrophages induced by the SF SS and SF-SS were similar with that of cotton gauze. *In vivo*, the amount of TNF-α from mice serum of SF SS, SF-SS, SF SS Exo or SF-SS Exo treatment groups was lower than that of the cotton gauze. The difference between the *in vitro* and *in vivo* effect may be due to the presence of various types of macrophages *in vivo*, as well as the different microenvironment of macrophages of *in vivo* and *in vitro*. In addition, CD68 immunofluorescence staining of the injured skin treated by the SF SS and SF-SS hydrogel sponges for 3 days showed similar results to that of the TNF-α (Figures 7A,B).

## Discussion

In our study, the beneficial effects were more significant in the experimental group treated by exosomes encapsulated in SF-SS hydrogel compared to SF SS. The beneficial effects may be explained by the fact that exosomes could increase the fibroblast cell viability *in vitro* (Figure 1D). In addition, Exo encapsulated in SF-SS hydrogels could promote vascular growth (Figure 4) and inhibit the inflammatory response (Figures 7A,B), suggesting that these hydrogels are promising biomaterials that can be used to enhance wound repair.

Exosomes are readily accessible via biological fluids for diagnosis and reflect the disease progression and prognosis, such as in cancer (29), cardiovascular, brain and kidney diseases (33–37). In addition, exosomes involve in regulating multiple biological process, such as reproduction and development, immune response, infection, neurodegeneration, cancer and cardiovascular disease through mediating cell communication (38–40). More importantly, exosomes derived from different kind of cells can treat multiple diseases including ischemic disease (41), cancer (42–44) and inflammatory bowel disease (45). In this study, we also found beneficial function of exosomes derived from UMSC in wound repair.

To improve the retention time of exosomes *in vivo*, we introduced hydrogel formed by SS and SF. It has been found that undegummed native silk can induce a severe immunoglobulin E mediated allergy or inflammation, delaying the wound healing process (46). However, there are some conflicting reports regarding the possible immunogenicity of silk proteins. SS has been considered as the element in the silk that triggers immune responses (47). However, recent studies showed that the immunogenicity of SS is extremely low. Furthermore, SS promotes the proliferation of vascular endothelial progenitor cells, endothelial cells, and hair follicle stem cells by increasing the transcription of VEGFa (48). Thus, SS has been used to treat myocardial infarction (49), skin wound (50), and bone injury (51–53). During the purification of silk sericin, the majority of small molecule impurities such as waxes, sugars and fats are expected to be removed during dialysis (46, 54), which may indicate that impurities in sericin is the cause for immune response. Another study showed that synergistic effect of crystalline fibroin coated with sericin protein and lipopolysaccharides, trigging significant release of TNF-α from macrophages, which indicates that sericin mediated inflammation activation is dependent on the association with core fibroin fibers (55). Even though there are different views on the source of the immune response, SF and SS hydrogel prepared in our article avoid the potential impurities and natural interaction of sericin and fibroin fibers during the purification and gelling process, which also showed very low immune response.

Previous studies showed that different extraction methods of sericin have different effects on protein integrity, structure and functional properties. Conventional harsh method with high heat and alkali can lead to degradation of sericin into low molecular weights polypeptides, make it difficult to cross-link into hydrogel (23). While gentle LiBr extraction method can produce a relative intact protein profile of sericin and forms a hydrogel that possesses excellent cell-adhesive capability, and promoting proliferation and long-term survival of various types of cells (32, 49). These can explain to a certain extent that SF-SS hydrogel has a short gelling time. SF hydrogel (14, 56) or SS hydrogel (9, 57) have been used to deliver cells and drugs due to the excellent mechanical properties of SF or bioactivity activity of SS. Composite hydrogels such as SF and chitosan hydrogel (58), SF and collagen hydrogel (59), SF and agar hydrogel (60), SF and poly (vinyl alcohol) hydrogel (61), have been used in the past to overcome some of the shortcomings associated with hydrogels formed by single biomaterial. SF and SS have emerged as promising biomaterial for tissue repair because they can be easily acquired and have low immunogenicity (48, 61, 62).

Mechanical properties of extracellular environment (ECM) are intimately related with cell behaviors. Those mechanical signals can be transfer by transmembrane receptors, cytoskeleton or nuclear skeleton, thus regulating the gene expression. Matrix elasticity has been shown to involve in cell survival, proliferation and differentiation (63, 64). Suitable mechanical properties of ECM are critical for the homeostasis of cell or tissue. Exception of bone, most of human tissues are soft, reflected by the value of compressive moduli (Young's moduli) ranging from 1 to 200 kPa (65–67). A better hydrogel should provide similar mechanical properties of certain tissue. As described before, the Young's moduli of SF hydrogel at concentration of 1.5–4% (w/v) ranges from 6.41 ± 0.47 kPa to 63.98 ± 2.42 kPa, which is consistent with a biologically relevant stiffness similar to native vasculature (68). Other studies showed that native silk fibers and fibroin hydrogel at concentrations of 4–15% (w/v) had the value of Young's moduli larger than 400 kPa, which is more suitable for stiff or semi-stiff implants, rather than for regeneration of soft organs (25). The Young's moduli of SS and polyvinyl alcohol hydrogel is only 20 Pa, which is much lower than that of most tissues (22). Thus, introducing SS into the fibroin hydrogel can adjust the compressive moduli to get a complex hydrogel with appropriate mechanical properties for wound repair.

Recent studies show that the hydrogels in encapsulating stem cells shape cell status, improve cells retention time and involve in tissue repair process (69, 70). In addition, recent works demonstrate that the beneficial functions of stem cells in tissue regeneration are largely through paracrine manner, rather than the direct differentiation of the implanted cell (71, 72). During all the secretome, exosomes are regarded as important components to involve in the cell communication and transfer functional molecules for tissue regeneration. We also showed that introducing exosomes derived from UMSCs into SF and SS mixed hydrogel promoted wound repair.

## Conclusion

In our study, we found that the freeze-dried composite hydrogel formed by SF and SS can be used to enhance wound repair with low immune response. In addition, the beneficial effects of these hydrogel were improved in the presence of UMSC-Exo. In conclusion, SF and SS mixed hydrogel can serve as a good substitute to wound dressing and can be used to deliver exosomes.

## Data Availability Statement

The raw data supporting the conclusions of this article will be made available by the authors, without undue reservation.

## Ethics Statement

The animal study was reviewed and approved by Laboratory Animal Care and Use Committee of Soochow University.

## Author Contributions

YL and CH conceptualized the study, interpreted data, and wrote the manuscript. CH, FL, and YZ performed most of the experiments and statistical analyses. WC, WL, FD, LL, and CW made intellectual contributions in data interpretations. All authors contributed to the article and approved the submitted version.

## Conflict of Interest

The authors declare that the research was conducted in the absence of any commercial or financial relationships that could be construed as a potential conflict of interest.

## Publisher's Note

All claims expressed in this article are solely those of the authors and do not necessarily represent those of their affiliated organizations, or those of the publisher, the editors and the reviewers. Any product that may be evaluated in this article, or claim that may be made by its manufacturer, is not guaranteed or endorsed by the publisher.

## References

[1] Rani S Ritter T. The exosome - a naturally secreted nanoparticle and its application to wound healing. Adv Mater. (2016) 28:5542–52. 10.1002/adma.201504009 26678528

[2] Tottoli EM Dorati R Genta I Chiesa E Pisani S Conti B. Skin wound healing process and new emerging technologies for skin wound care and regeneration. Pharmaceutics. (2020) 12:735. 10.3390/pharmaceutics12080735 32764269 PMC7463929

[3] Zhang B Wang M Gong A Zhang X Wu X Zhu Y. HucMSC-exosome mediated-wnt4 signaling is required for cutaneous wound healing. Stem Cells. (2015) 33:2158–68. 10.1002/stem.1771 24964196

[4] Leoni G Neumann PA Kamaly N Quiros M Nishio H Jones HR. Annexin A1-containing extracellular vesicles and polymeric nanoparticles promote epithelial wound repair. J Clin Invest. (2015) 125:1215–27. 10.1172/JCI76693 25664854 PMC4362251

[5] Sjoqvist S Ishikawa T Shimura D Kasai Y Imafuku A Bou-Ghannam S. Exosomes derived from clinical-grade oral mucosal epithelial cell sheets promote wound healing. J Extracell Vesicles. (2019) 8:1565264. 10.1080/20013078.2019.1565264 30719240 PMC6346716

[6] Zhang W Bai X Zhao B Li Y Zhang Y Li Z. Cell-free therapy based on adipose tissue stem cell-derived exosomes promotes wound healing via the PI3K/Akt signaling pathway. Exp Cell Res. (2018) 370:333–42. 10.1016/j.yexcr.2018.06.035 29964051

[7] Kim S Lee SK Kim H Kim TM. Exosomes secreted from induced pluripotent stem cell-derived mesenchymal stem cells accelerate skin cell proliferation. Int J Mol Sci. (2018) 19:3119. 10.3390/ijms19103119 30314356 PMC6213597

[8] Xu N Wang L Guan J Tang C He N Zhang W. Wound healing effects of a Curcuma zedoaria polysaccharide with platelet-rich plasma exosomes assembled on chitosan/silk hydrogel sponge in a diabetic rat model. Int J Biol Macromol. (2018) 117:102–7. 10.1016/j.ijbiomac.2018.05.066 29772339

[9] Farokhi M Mottaghitalab F Fatahi Y Khademhosseini A Kaplan DL. Overview of silk fibroin use in wound dressings. Trends Biotechnol. (2018) 36:907–22. 10.1016/j.tibtech.2018.04.004 29764691

[10] Lamboni L Gauthier M Yang G Wang Q. Silk sericin: a versatile material for tissue engineering and drug delivery. Biotechnol Adv. (2015) 33:1855–67. 10.1016/j.biotechadv.2015.10.014 26523781

[11] Chouhan D Lohe TU Samudrala PK Mandal BB. In situ forming injectable silk fibroin hydrogel promotes skin regeneration in full thickness burn wounds. Adv Healthc Mater. (2018) 7:31. 10.1002/adhm.201801092 30379407

[12] Ciocci M Cacciotti I Seliktar D Melino S. Injectable silk fibroin hydrogels functionalized with microspheres as adult stem cells-carrier systems. Int J Biol Macromol. (2018) 108:960–71. 10.1016/j.ijbiomac.2017.11.013 29113887

[13] Fernández-García L Marí-Buyé N Barios JA Madurga R Elices M Pérez-Rigueiro J. Safety and tolerability of silk fibroin hydrogels implanted into the mouse brain. Acta Biomater. (2016) 45:262–75. 10.1016/j.actbio.2016.09.003 27592819

[14] Wang X Ding Z Wang C Chen X Xu H Lu Q. Bioactive silk hydrogels with tunable mechanical properties. J Mater Chem B. (2018) 6:2739–46. 10.1039/C8TB00607E 30345058 PMC6191054

[15] Yan Y Cheng B Chen K Cui W Qi J Li X. Enhanced osteogenesis of bone marrow-derived mesenchymal stem cells by a functionalized silk fibroin hydrogel for bone defect repair. Adv Healthc Mater. (2019) 8:28. 10.1002/adhm.201801043 30485718

[16] Hu J Lu Y Cai L Owusu-Ansah KG Xu G Han F. Functional compressive mechanics and tissue biocompatibility of an injectable SF/PU hydrogel for nucleus pulposus replacement. Sci Rep. (2017) 7:017–02497. 10.1038/s41598-017-02497-3 28539658 PMC5443820

[17] Wu J Zheng K Huang X Liu J Liu H Boccaccini AR. Thermally triggered injectable chitosan/silk fibroin/bioactive glass nanoparticle hydrogels for in-situ bone formation in rat calvarial bone defects. Acta Biomater. (2019) 91:60–71. 10.1016/j.actbio.2019.04.023 30986530

[18] Zheng A Cao L Liu Y Wu J Zeng D Hu L. Biocompatible silk/calcium silicate/sodium alginate composite scaffolds for bone tissue engineering. Carbohydr Polym. (2018) 199:244–55. 10.1016/j.carbpol.2018.06.093 30143127

[19] Cao TT Zhang YQ. Processing and characterization of silk sericin from Bombyx mori and its application in biomaterials and biomedicines. Mater Sci Eng C Mater Biol Appl. (2016) 61:940–52. 10.1016/j.msec.2015.12.082 26838924

[20] Ahsan F Ansari TM Usmani S Bagga P. An insight on silk protein sericin: from processing to biomedical application. Drug Res. (2018) 68:317–27. 10.1055/s-0043-121464 29132177

[21] Kamalathevan P Ooi PS Loo YL. Silk-based biomaterials in cutaneous wound healing: a systematic review. Adv Skin Wound Care. (2018) 31:565–73. 10.1097/01.ASW.0000546233.35130.a9 30475285

[22] Siritienthong T Ratanavaraporn J Aramwit P. Development of ethyl alcohol-precipitated silk sericin/polyvinyl alcohol scaffolds for accelerated healing of full-thickness wounds. Int J Pharm. (2012) 439:175–86. 10.1016/j.ijpharm.2012.09.043 23022662

[23] Kunz RI Brancalhao RM Ribeiro LF Natali MR. Silkworm sericin: properties and biomedical applications. Biomed Res Int. (2016) 8175701:14. 10.1155/2016/8175701 27965981 PMC5124675

[24] Napavichayanun S Ampawong S Harnsilpong T Angspatt A Aramwit P. Inflammatory reaction, clinical efficacy, and safety of bacterial cellulose wound dressing containing silk sericin and polyhexamethylene biguanide for wound treatment. Arch Dermatol Res. (2018) 310:795–805. 10.1007/s00403-018-1871-3 30302557

[25] Leal-Egaña A Scheibel T. Silk-based materials for biomedical applications. Biotechnol Appl Biochem. (2010) 55:155–67. 10.1042/BA20090229 20222871

[26] Lim KS Kundu J Reeves A Poole-Warren LA Kundu SC Martens PJ. The influence of silkworm species on cellular interactions with novel PVA/silk sericin hydrogels. Macromol Biosci. (2012) 12:322–32. 10.1002/mabi.201100292 22493796

[27] Han C Zhou J Liang C Liu B Pan X Zhang Y. Human umbilical cord mesenchymal stem cell derived exosomes encapsulated in functional peptide hydrogels promote cardiac repair. Biomater Sci. (2019) 15:2920–33. 10.1039/C9BM00101H 31090763

[28] Han C Zhou J Liu B Liang C Pan X Zhang Y. Delivery of miR-675 by stem cell-derived exosomes encapsulated in silk fibroin hydrogel prevents aging-induced vascular dysfunction in mouse hindlimb. Mater Sci Eng C Mater Biol Appl. (2019) 99:322–32. 10.1016/j.msec.2019.01.122 30889706

[29] Casson J Davies OG Smith CA Dalby MJ Berry CC. Mesenchymal stem cell-derived extracellular vesicles may promote breast cancer cell dormancy. J Tissue Eng. (2018) 9:2041731418810093. 10.1177/2041731418810093 30627418 PMC6311537

[30] Rockwood DN Preda RC Yucel T Wang X Lovett ML Kaplan DL. Materials fabrication from Bombyx mori silk fibroin. Nat Protoc. (2011) 6:1612–31. 10.1038/nprot.2011.379 21959241 PMC3808976

[31] Shao L Zhang Y Lan B Wang J Zhang Z Zhang L. MiRNA-sequence indicates that mesenchymal stem cells and exosomes have similar mechanism to enhance cardiac repair. Biomed Res Int. (2017) 4150705:22. 10.1155/2017/4150705 28203568 PMC5292186

[32] Wang Z Zhang Y Zhang J Huang L Liu J Li Y. Exploring natural silk protein sericin for regenerative medicine: an injectable, photoluminescent, cell-adhesive 3D hydrogel. Sci Rep. (2014) 4:7064. 10.1038/srep07064 25412301 PMC4238302

[33] Alvarez-Erviti L Seow Y Schapira AH Gardiner C Sargent IL Wood MJ. Lysosomal dysfunction increases exosome-mediated alpha-synuclein release and transmission. Neurobiol Dis. (2011) 42:360–7. 10.1016/j.nbd.2011.01.029 21303699 PMC3107939

[34] Cheng M Yang J Zhao X Zhang E Zeng Q Yu Y. Circulating myocardial microRNAs from infarcted hearts are carried in exosomes and mobilise bone marrow progenitor cells. Nat Commun. (2019) 10:019–08895. 10.1038/s41467-019-08895-7 30814518 PMC6393447

[35] Nilsson J Skog J Nordstrand A Baranov V Mincheva-Nilsson L Breakefield XO. Prostate cancer-derived urine exosomes: a novel approach to biomarkers for prostate cancer. Br J Cancer. (2009) 100:1603–7. 10.1038/sj.bjc.6605058 19401683 PMC2696767

[36] Rajendran L Honsho M Zahn TR Keller P Geiger KD Verkade P. Alzheimer's disease beta-amyloid peptides are released in association with exosomes. Proc Natl Acad Sci U S A. (2006) 103:11172–7. 10.1073/pnas.0603838103 16837572 PMC1544060

[37] Zhou H Pisitkun T Aponte A Yuen PS Hoffert JD Yasuda H. Exosomal Fetuin-A identified by proteomics: a novel urinary biomarker for detecting acute kidney injury. Kidney Int. (2006) 70:1847–57. 10.1038/sj.ki.5001874 17021608 PMC2277342

[38] Kalluri R LeBleu VS. The biology, function, and biomedical applications of exosomes. Science. (2020) 367:6478. 10.1126/science.aau6977 32029601 PMC7717626

[39] Kita S Maeda N Shimomura I. Interorgan communication by exosomes, adipose tissue, and adiponectin in metabolic syndrome. J Clin Invest. (2019) 129:4041–9. 10.1172/JCI129193 31483293 PMC6763291

[40] Sadri Nahand J Moghoofei M Salmaninejad A Bahmanpour Z Karimzadeh M Nasiri M. Pathogenic role of exosomes and microRNAs in HPV-mediated inflammation and cervical cancer: A review. Int J Cancer. (2020) 146:305–20. 10.1002/ijc.32688 31566705 PMC6999596

[41] Ong SG Lee WH Huang M Dey D Kodo K Sanchez-Freire V. Cross talk of combined gene and cell therapy in ischemic heart disease: role of exosomal microRNA transfer. Circulation. (2014) 130:007917. 10.1161/CIRCULATIONAHA.113.007917 25200057 PMC4862832

[42] Bu H He D He X Wang K. Exosomes: isolation, analysis, and applications in cancer detection and therapy. Chembiochem. (2019) 20:451–61. 10.1002/cbic.201800470 30371016

[43] Elsherbini A Bieberich E. Ceramide and exosomes: a novel target in cancer biology and therapy. Adv Cancer Res. (2018) 140:121–54. 10.1016/bs.acr.2018.05.004 30060807 PMC6109973

[44] Melo SA Luecke LB Kahlert C Fernandez AF Gammon ST Kaye J. Glypican-1 identifies cancer exosomes and detects early pancreatic cancer. Nature. (2015) 523:177–82. 10.1038/nature14581 26106858 PMC4825698

[45] Zhang H Wang L Li C Yu Y Yi Y Wang J. Exosome-Induced Regulation in Inflammatory Bowel Disease. Front Immunol. (2019) 10:1464. 10.3389/fimmu.2019.01464 31316512 PMC6611439

[46] Zakeri Siavashani A Mohammadi J Maniura-Weber K Senturk B Nourmohammadi J Sadeghi B. Silk based scaffolds with immunomodulatory capacity: anti-inflammatory effects of nicotinic acid. Biomater Sci. (2019) 8:148–62. 10.1039/C9BM00814D 31663545

[47] Thurber AE Omenetto FG Kaplan DL. *In vivo* bioresponses to silk proteins. Biomaterials. (2015) 71:145–57. 10.1016/j.biomaterials.2015.08.039 26322725 PMC4573254

[48] Jiao Z Song Y Jin Y Zhang C Peng D Chen Z. *In Vivo* characterizations of the immune properties of sericin: an ancient material with emerging value in biomedical applications. Macromol Biosci. (2017) 17:17. 10.1002/mabi.201700229 29045024

[49] Song Y Zhang C Zhang J Sun N Huang K Li H. An injectable silk sericin hydrogel promotes cardiac functional recovery after ischemic myocardial infarction. Acta Biomater. (2016) 41:210–23. 10.1016/j.actbio.2016.05.039 27262742

[50] Qi C Xu L Deng Y Wang G Wang Z Wang L. Sericin hydrogels promote skin wound healing with effective regeneration of hair follicles and sebaceous glands after complete loss of epidermis and dermis. Biomater Sci. (2018) 6:2859–70. 10.1039/C8BM00934A 30259043

[51] Griffanti G Jiang W Nazhat SN. Bioinspired mineralization of a functionalized injectable dense collagen hydrogel through silk sericin incorporation. Biomater Sci. (2019) 7:1064–77. 10.1039/C8BM01060A 30629053

[52] Yang M Mandal N Shuai Y Zhou G Min S Zhu L. Mineralization and biocompatibility of Antheraea pernyi (A. pernyi) silk sericin film for potential bone tissue engineering. Biomed Mater Eng. (2014) 24:815–24. 10.3233/BME-130873 24211968

[53] Zhong Q Li W Su X Li G Zhou Y Kundu SC. Degradation pattern of porous CaCO3 and hydroxyapatite microspheres in vitro and in vivo for potential application in bone tissue engineering. Colloids Surf B Biointerfaces. (2016) 143:56–63. 10.1016/j.colsurfb.2016.03.020 26998866

[54] Ak F Oztoprak Z Karakutuk I Okay O. Macroporous silk fibroin cryogels. Biomacromolecules. (2013) 14:719–27. 10.1021/bm3018033 23360211

[55] Panilaitis B Altman GH Chen J Jin HJ Karageorgiou V Kaplan DL. Macrophage responses to silk. Biomaterials. (2003) 24:3079–85. 10.1016/S0142-9612(03)00158-3 12895580

[56] Pritchard EM Kaplan DL. Silk fibroin biomaterials for controlled release drug delivery. Expert Opin Drug Deliv. (2011) 8:797–811. 10.1517/17425247.2011.568936 21453189

[57] Wang Y Cai R Tao G Wang P Zuo H Zhao P. A novel AgNPs/Sericin/Agar film with enhanced mechanical property and antibacterial capability. Molecules. (2018) 23:1821. 10.3390/molecules23071821 30041405 PMC6100604

[58] Adali T Kalkan R Karimizarandi L. The chondrocyte cell proliferation of a chitosan/silk fibroin/egg shell membrane hydrogels. Int J Biol Macromol. (2019) 124:541–7. 10.1016/j.ijbiomac.2018.11.226 30496865

[59] Buitrago JO Patel KD El-Fiqi A Lee JH Kundu B Lee HH. Silk fibroin/collagen protein hybrid cell-encapsulating hydrogels with tunable gelation and improved physical and biological properties. Acta Biomater. (2018) 69:218–33. 10.1016/j.actbio.2017.12.026 29410166

[60] Tyeb S Kumar N Kumar A Verma V. Flexible agar-sericin hydrogel film dressing for chronic wounds. Carbohydr Polym. (2018) 200:572–82. 10.1016/j.carbpol.2018.08.030 30177201

[61] Tao H Kaplan DL Omenetto FG. Silk materials–a road to sustainable high technology. Adv Mater. (2012) 24:2824–37. 10.1002/adma.201104477 22553118

[62] Zhao Z Li Y Xie MB. Silk fibroin-based nanoparticles for drug delivery. Int J Mol Sci. (2015) 16:4880–903. 10.3390/ijms16034880 25749470 PMC4394455

[63] Helenius J Heisenberg CP Gaub HE Muller DJ. Single-cell force spectroscopy. J Cell Sci. (2008) 121:1785–91. 10.1242/jcs.030999 18492792

[64] Vogel V. Mechanotransduction involving multimodular proteins: converting force into biochemical signals. Annu Rev Biophys Biomol Struct. (2006) 35:459–88. 10.1146/annurev.biophys.35.040405.102013 16689645

[65] Chang G Kim HJ Vunjak-Novakovic G Kaplan DL Kandel R. Enhancing annulus fibrosus tissue formation in porous silk scaffolds. J Biomed Mater Res A. (2010) 92:43–51. 10.1002/jbm.a.32326 19165797

[66] Hendriks FM Brokken D Oomens CW Bader DL Baaijens FP. The relative contributions of different skin layers to the mechanical behavior of human skin *in vivo* using suction experiments. Med Eng Phys. (2006) 28:259–66. 10.1016/j.medengphy.2005.07.001 16099191

[67] Salameh N Peeters F Sinkus R Abarca-Quinones J Annet L Ter Beek LC. Hepatic viscoelastic parameters measured with MR elastography: correlations with quantitative analysis of liver fibrosis in the rat. J Magn Reson Imaging. (2007) 26:956–62. 10.1002/jmri.21099 17896384

[68] Floren M Bonani W Dharmarajan A Motta A Migliaresi C Tan W. Human mesenchymal stem cells cultured on silk hydrogels with variable stiffness and growth factor differentiate into mature smooth muscle cell phenotype. Acta Biomater. (2016) 31:156–66. 10.1016/j.actbio.2015.11.051 26621695 PMC4728007

[69] Chaudhuri O Gu L Klumpers D Darnell M Bencherif SA Weaver JC. Hydrogels with tunable stress relaxation regulate stem cell fate and activity. Nat Mater. (2016) 15:326–34. 10.1038/nmat4489 26618884 PMC4767627

[70] Lee JH Kim HW. Emerging properties of hydrogels in tissue engineering. J Tissue Eng. (2018) 9:2041731418768285. 10.1177/2041731418768285 29623184 PMC5881958

[71] Alcaraz MJ Compañ A Guillén MI. Extracellular vesicles from mesenchymal stem cells as novel treatments for musculoskeletal diseases. Cells. (2019) 9:98. 10.3390/cells9010098 31906087 PMC7017209

[72] Andia I Maffulli N Burgos-Alonso N. Stromal vascular fraction technologies and clinical applications. Expert Opin Biol Ther. (2019) 19:1289–305. 10.1080/14712598.2019.1671970 31544555

